# Latent layers in social networks and their implications for comparative analyses

**DOI:** 10.1093/beheco/araf113

**Published:** 2025-09-29

**Authors:** Delphine De Moor, Jordan D A Hart, Daniel W Franks, Lauren J N Brent, Matthew J Silk, Josefine B Brask

**Affiliations:** Centre for Research in Animal Behaviour, University of Exeter, Perry Rd, Exeter EX4 4QG, United Kingdom; Department of Primate Behavior and Evolution, Max Planck Institute for Evolutionary Anthropology, Deutscher Platz 6, Leipzig 04103, Germany; Centre for Research in Animal Behaviour, University of Exeter, Perry Rd, Exeter EX4 4QG, United Kingdom; Department of Biology, University of York, York YO10 5DD, United Kingdom; Centre for Research in Animal Behaviour, University of Exeter, Perry Rd, Exeter EX4 4QG, United Kingdom; Institute of Ecology and Evolution, School of Biological Sciences, University of Edinburgh, Charlotte Auerbach Road, Edinburgh EH9 3FL, United Kingdom; CEFE, Université de Montpellier, CNRS, EPHE, IRD, UMR 5175, Montpellier 34090, France; Copenhagen Center for Social Data Science (SODAS), University of Copenhagen, Øster Farimagsgade 5, Copenhagen 1357, Denmark; Section for Ecology and Evolution, Department of Biology, University of Copenhagen, Universitetsparken 15, Copenhagen 2100, Denmark

**Keywords:** Bayesian models, comparative analysis, generative models, social evolution, social networks, social system

## Abstract

Animal social systems are remarkably diverse, ranging from solitary individuals to well-connected cooperative groups. Understanding the drivers of this variation is a key question in behavioral ecology and has been the focus of numerous studies linking social structure to ecological, demographic, and life history patterns within groups, population, and species. Equipped with this information, researchers are now turning to investigations that are comparative in nature. However, comparing social networks remains a considerable logistical and analytical challenge. Here, we present the *latent layers framework*, which outlines how *observed social networks* are linked to the 2 underlying latent networks that are of interest for most research questions: the *realised social network* (the actual pattern of social interactions), and the *social preference network* driving these interactions. This conceptual framework provides a clear and unified approach to understand when and why differences in network properties and sampling protocols can introduce discrepancies between observed and latent networks, potentially biasing or confounding statistical inference. We then use this conceptual framework to outline some of the central challenges to comparing animal social networks, describe why and how they create challenges for comparative analyses, and suggest potential directions for solutions. The *latent layers framework* can help researchers to identify networks they can (or cannot) compare. In doing so, this framework facilitates advances in comparative social network studies with the potential to generate new and important insights into the ecological and evolutionary drivers of variation in social structure across the animal kingdom.

## Teaser

Animal social networks vary widely. What drives this variation? One key way to find out is by comparing species that live in different environments and have different lifestyles. But such comparisons are analytically challenging. In this review, we introduce the ‘latent layers framework’ to explain when and why problems occur. We also explore five major challenges in comparing social networks and offer solutions to move the field forward.

## Introduction

The animal kingdom features a remarkable diversity of social systems, from solitary individuals to well-connected cooperative groups (Clutton-Brock 2016; Rubenstein and Abbot 2017). Yet, our understanding of the ecological and evolutionary causes and consequences of this diversity remains incomplete (Kurvers et al. 2014; Kappeler 2019). Comparing animal populations facing distinct environmental challenges can provide insights into the influence of ecological variables such as predation and food availability on social patterns (Krause and Ruxton 2002; Lukas and Clutton-Brock 2018; Barsbai et al. 2021; Bonnell et al. 2022), and reciprocally, how these social patterns impact ecological factors, such as pathogen transmission (Bansal et al. 2007; White et al. 2017; Albery et al. 2021). Comparative analyses can also reveal how social systems evolve alongside life history traits (Silk and Hodgson 2021) and interact with demographic mechanisms (Shizuka and Johnson 2020; Clements et al. 2022).

As a result of countless efforts to collect social data (Clutton-Brock 2021; Sheldon et al. 2022), numerous animal social datasets exist, capturing social structure across diverse species and environments. With the emergence of databases bringing together these data across taxa (eg MacaqueNet, De Moor et al. 2025; Animal Social Network Repository (ASNR), Sah et al. 2019; DomArchive, Strauss et al. 2022), attention has turned to the statistical methods with which such data can be compared (Shizuka and McDonald 2015; Hobson et al. 2019; Ellis et al. 2021; Albery et al. 2024).

Comparative social network analysis offers a holistic approach to draw inference about the drivers and consequences of animal social structure (Pinter-Wollman et al. 2013; Shizuka and McDonald 2015; Croft et al. 2016; Hobson et al. 2019; Webber and Vander Wal 2019; Albery et al. 2024). By explicitly representing social structure as an emergent property of social interactions between individuals (Hinde 1976), social network analysis can be used to ask questions about social evolution at the level of individuals, dyads, social groups, and populations. Various insights into animal societies can therefore be gained by either directly comparing the entire networks as objects themselves (eg correlating entire network matrices) or after compressing networks into summary measures and statistics (eg comparing global, dyadic and/or individual network metrics or other summary statistics; McDonald and Shizuka 2013; Hobson et al. 2019). However, despite the potential value of comparative social network analysis, only a relatively small body of literature compares social networks across species and taxonomic groups (Albery et al. 2024). A major reason for the lack of such studies is the viability of comparing networks that are generated using different methodologies, and that may diverge widely in key properties such as network size and behavior types (Faust and Skvoretz 2002; Pinter-Wollman et al. 2013; Ogino et al. 2023).

Here, we offer an overview of 5 key challenges that create disparities between social networks and provide guidance on what to consider when designing comparative analyses to minimize these challenges. A common theme to these challenges is that the observed networks we compare are most often different from the underlying, latent (ie unobservable), networks we want to make inferences about. We therefore begin by introducing the *latent layers framework* that represents how different biological and observational processes interact to influence the social structures we observe. Our aim is to outline these issues in a way that helps behavioral ecologists in making meaningful and informed comparisons of social structures. A clear understanding of how these processes contribute to variation in observed social networks will help researchers to make principled decisions on how best to compare network, regardless of the nature of their comparison (eg comparing networks across populations or across time in the same population). While we introduce the *latent layers framework* in the context of comparing networks, it contains valuable concepts for all social network analyses, be they comparative or not.

## The latent layers framework

One key issue in network analysis is that observed networks often do not directly, or exclusively, correspond to the biological phenomenon of interest (Brugere et al. 2018; Kawam et al. 2025). This lack of correspondence is due to 2 primary reasons. Firstly, the social networks we quantify are usually based on only a subset of interactions—those that have been recorded—and an observed network is therefore an estimation, not exact representation, of the complete or “real” patterning of interactions (Handcock and Gile 2010; Shizuka and McDonald 2015). Secondly, even when all interactions that happen in a group are recorded, they are unlikely to correspond directly to individual preferences in those interactions because of constraints that hinder individuals in realising their preferred relationships. For example, if all individuals in a group prefer high-ranking individuals as partners, only a subset of those individuals may have that preference realized because the time high-ranking individuals have available to socialize is limited (Seyfarth 1977). Instead, some individuals might end up interacting with their second, third, or even last choice of partner.

**Fig. 1.**
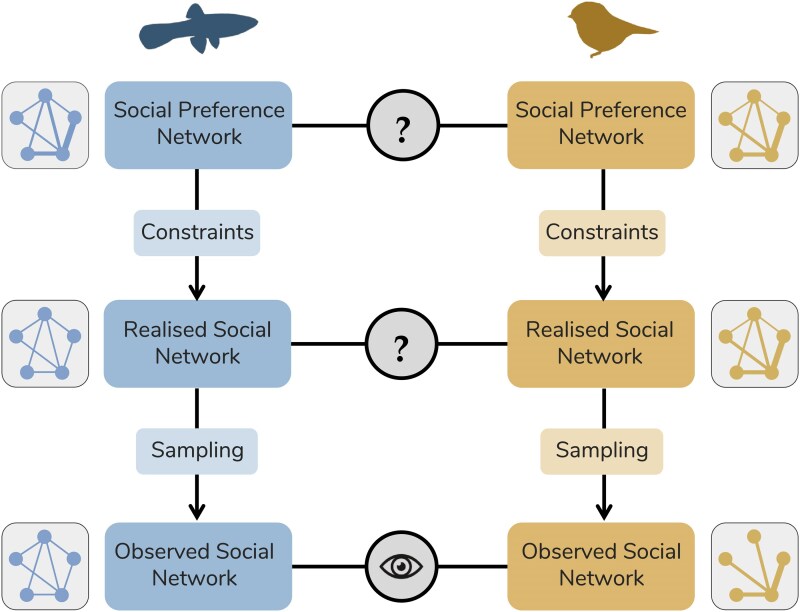
The latent layers framework. Social structure can be considered as a hierarchy of networks: an observed social network to which researchers have access, and 2 layers of latent networks—the realized social network and the social preference network. When we compare networks across species (eg here a fish and a bird), we typically compare observed social networks (the eye icon), but our research questions typically concern differences between realized social networks or between social preference networks (the question mark icons). To ensure that analyses of the observed networks accurately reflect the latent networks of interest, it is important to consider the factors along the path from the relevant question mark to the eye that could result in differences between the observed and the realized and social preference networks. To illustrate this, we show an example with small networks. In this example, the 2 species have identical social preference networks, but they end up having very different observed social networks, due to the effects of constraints and sampling biases. The fish species has limited time available to socialize, which constrains individuals in establishing strong links. The realized social network is therefore very different from the social preference network. Sampling of the fish species is unbiased, so the observed social network corresponds to the realized social network. In contrast, the bird species is not affected by constraints, and the realized social network therefore corresponds to the social preference network. However, sampling of the bird species is biased, with more gregarious individuals being more visible to the observers. The observed social network is therefore very different from the realized social network. If the effects of constraints and sampling biases are not considered, researchers would erroneously conclude that the 2 species have different types of social preferences, and that the realized social networks differ more than they actually do.

To provide a structured way of thinking about this, we present the *latent layers framework*, which represents social structure as a hierarchy of networks: an *observed social network*, the *realised social network* and the *social preference network* (Fig. 1). The realized social network and the social preference network are latent and cannot be directly observed, but they can be inferred from the observed social interactions. An *observed network* represents a sample of the *realised network*. The *realised network* is the actual pattern of all interactions or associations between individuals. In turn, the *realised network* is itself a (likely partial) realization of the individuals’ social preferences. These preferences can also be represented as a network (eg a directed network representing the strength of preference that each individual has for others for a given behavior, see Box 2): the *social preference network*.

Mismatches between an *observed network* and the 2 latent networks (or indeed between the 2 latent networks) are the result of 2 main processes: *sampling biases* and *constraints* (Fig. 1). Common examples of *sampling biases* include cases where certain individuals or interactions are better sampled than others (Altmann 1974; Bateson and Martin 2021). For example, a researcher using biologgers to record proximity between pairs of animals may not be able to afford to put a biologger on every individual, or a researcher visually observing social interactions cannot simultaneously watch all individuals at once. *Constraints* include factors that prevent individuals from realizing their social preferences. These constraints may come from different sources, such as incompatible preferences between potential partners, where one individual may wish to interact but the other does not, preventing the preferred relationship from forming. Other examples include spatial constraints, where individuals are separated by physical barriers or large distances that prevent access to preferred partners, and social factors such as dominance and kinship structures, which limit opportunities for interaction (Webber and Vander Wal 2018; Fisher et al. 2021).

Research questions are almost always about the latent network layers rather than the *observed network* (Lundberg et al. 2021). The *social preference network* is usually the level of interest when researchers seek to understand the causes of social behaviors, such as how kinship, age and sex impact partner choice, or the role of life history, ecology, or the social environment in shaping the types of social relationships individuals form (Smith 2014; Snyder-Mackler et al. 2016; Chakrabarti et al. 2020; De Moor et al. 2020; Hobson, Monster et al. 2021; Silk and Hodgson 2021; Siracusa et al. 2022). On the other hand, the *realised social network* is usually the level of interest for investigations into the consequences of social behaviors, such as the influence of social structure on disease or information transmission (Aplin et al. 2012; Silk and Fefferman 2021; Collier et al. 2022) and on fitness outcomes (Ellis et al. 2017; Riehl and Strong 2018; Ellis et al. 2019; Strauss and Holekamp 2019; Sabol et al. 2020). However, analyses are typically run on the level of the *observed network* (Fig. 1). Understanding which latent network layer is of interest for a given question, and how an *observed network* relates to that latent layer is therefore essential for reliable social network analyses.

## Challenges of comparative social network analysis

Comparative social network analysis faces significant challenges due to the variability in how networks are constructed. Networks can be based on different behaviors, sampled using diverse data collection methods with varying degrees of effort, and sampled at different biological scales (Faust and Skvoretz 2002; Davis et al. 2018; Canteloup et al. 2020; Albery et al. 2024). These differences can create disparities between the *observed networks*, reflecting *sampling biases* and *constraints* rather than true differences, thus confounding comparative analysis (Shizuka and McDonald 2015; Gagliardi et al. 2023; Ogino et al. 2023). Recent methodological developments that treat observed interactions as the outcome of generative processes that can be modeled, allow researchers to explicitly account for sampling biases and constraints analytically by inferring the latent network layers based on the observed network; for further technical detail, see Box 1.

Box 1: Moving between layers Recent developments in Bayesian network analysis provide methods that allow us to attempt moving between the network layers depicted in Fig. 1 (De Bacco et al. 2023; Hart et al. 2023; Redhead et al. 2023; Kawam et al 2025).

These Bayesian models treat observed interactions as the outcome of a 2-step process: a generative process that gives rise to the latent realized network (eg reflecting social preferences, influenced by factors such as individual traits, dyadic relatedness, or environmental conditions) and an observation process that links the realized network to the observed data (influenced by sampling-related factors such as effort and biases in the visibility of individuals or behaviors). By explicitly modeling both processes, these models estimate the distribution of plausible latent social networks that could have produced the observed interactions, while accounting for constraints in realizing social preferences and sampling biases in observation. Similar approaches are commonly used in ecological Hidden Markov Models, for example, to estimate demographic states from capture–recapture data (Gimenez and Gaillard 2017; McClintock et al. 2020).

In essence, these generative modeling approaches estimate the latent network layers underlying social interaction patterns based on the observed data, while incorporating information about the processes that generate the *observed networks*. For example, starting from the observed fish and bird networks in our example (Fig. 1), we can incorporate our knowledge about the suspected constraints (limited time to socialize in the fish population) and sampling biases (overrepresentation of gregarious individuals in the bird population) when estimating distributions of possible social preference and realized networks that could underlie those observed social networks.

Generative modeling approaches can be used to quantify how well the observed network is expected to reflect the underlying latent network layers, which is translated into uncertainties in the estimated strength of dyadic connections (ie edge weights; Ross et al. 2023). For instance, within the BISoN framework (Hart et al. 2023), network metrics are calculated from draws of the Bayesian posterior distribution of the latent *realised network* edge weights, while accounting for sampling effort and structured influences where needed. Doing so generates a posterior distribution of network metric values (instead of a single value, or point estimate), therefore, explicitly including uncertainty in the metric estimates, where higher sampling effort leads to narrower distributions. Once network metric posteriors have been generated, they can be passed to downstream statistical analyses thereby carrying uncertainty forward into statistical analyses, such that networks with higher observation effort carry more weight on inference. In our example (Fig. 1), gregarious birds are observed more often than less gregarious ones. By using BISoN, this sampling bias is taken into account by narrower posterior distributions for the edge weights and network metrics of those more frequently observed individuals—indicating greater confidence in their estimated social positions, which will therefore carry more weight in downstream inference.

These methodological developments hold great promise for comparative social network analysis. They enable researchers to make inferences at the latent network level relevant to specific research questions, while also explicitly accounting for differences between networks that could potentially confound or bias comparisons (Fig. 1). However, moving between layers is still challenging, especially for systems where the generative social processes are poorly understood. Bayesian models that estimate latent network layers require causal inference, and so information on the *generative processes* giving rise to the *observed networks*, which include both biological (eg, social preferences and social and/or physical constraints in realizing those preferences) and observational (eg, sampling protocols and effort) factors (Pearl and Mackenzie 2018; Franks et al. 2025).

For instance, individual decisions regarding who to affiliate and fight with are likely driven by underlying rules based on characteristics of the individual, their potential social partners, and the broader social context (Hobson, Monster et al. 2021). Identifying what these factors are, and what needs to be controlled for statistically needs to be defined using causal inference methods such as Directed Acyclic Graphs (DAGs) that define the data generating process (Franks et al. 2025). Yet, understanding these generative processes—and the extent to which they are generalizable across taxa—is still very much in development in animal social network analysis (Hobson, Monster et al. 2021; Hobson, Silk et al. 2021; Brask et al. 2023). This is an area where significant methodological advancements are needed to fully enable reliable comparative social network analysis. The *latent layers* framework offers a clear and structured approach to understanding these generative processes. It makes explicit how the *observed networks* used in research are generated by underlying *social preferences* and are modulated by *constraints* and *sampling biases*.

Here, we consider 5 key challenges in comparative social network analysis: comparing networks that differ in (1) behavior type, (2) sampling method, (3) sampling effort, (4) network size, and (5) biological scale. We summarize these challenges in Table 1 and discuss in greater detail how these differences introduce *sampling biases* and *constraints*, generating discrepancies between the observed and latent networks and affecting the comparisons of observed networks. Additionally, we provide guidance on how best to handle each of these challenges, and—because the most appropriate solutions often depend on the study systems being used and the questions being asked—we build on the example above comparing a fish network to a bird network (Fig. 1) to make this guidance more concrete and practically useful. In our example, we are interested in comparing the social structure of the 2 species, asking whether individuals prefer to form many social relationships or a few strong ones. This question lies at the level of the social preference network, meaning that we need to consider both sampling biases and constraints. At the end of the section on each challenge, we explain how we deal with differences in the observed networks of the 2 species to make comparisons as robust as possible.

**Table 1.** Summary of 5 key challenges faced when comparing animal social networks.

Challenge	Description
Behavior type	Networks vary in the behaviors used to construct them. Whether networks based on different (or the same) behaviors can be compared depends on the biological functions of the behaviors in the given populations and the research question.
Sampling effort	Sampling effort can vary substantially between networks, influencing how reliably an *observed network* represents the *realised network*. Bayesian models that estimate the *realised network* from observed data can account for sampling effort as uncertainty in the estimated network properties (Box 1).
Sampling type	Networks constructed using various sampling methodologies pose 2 main challenges: (1) different sampling protocols generate different sampling biases, and (2) how the strength of dyadic connections (edge weights) are quantified may not be directly comparable. Recent methodological advancements, such as mixture models and Bayesian models that estimate the *realised* or *social preference network* from an *observed network* while explicitly accounting for sampling type, offer promising solutions (Box 1).
Network size	Networks can vary substantially in their size, which can influence network structure. Whether or not to account for network size depends on whether network size is central to the relationship between network structure and biological variables of interest (Fig. 2). If controlling for network size is warranted, doing so correctly can be challenging as it requires knowledge of the generative process underlying the network (Box 1), which determines how size impacts the network property of interest.
Network scale	Networks can be sampled at various scales, resulting in *observed networks* representing different subsets of *realised networks*. The scale of sampling significantly influences network structure, making networks sampled at different scales generally incomparable, particularly for global network properties. Bayesian models that impute missing data for networks sampled at smaller scales may provide a solution, but they necessitate an understanding of the generative process underlying the network at the larger scale (Box 1).

**Fig. 2.**
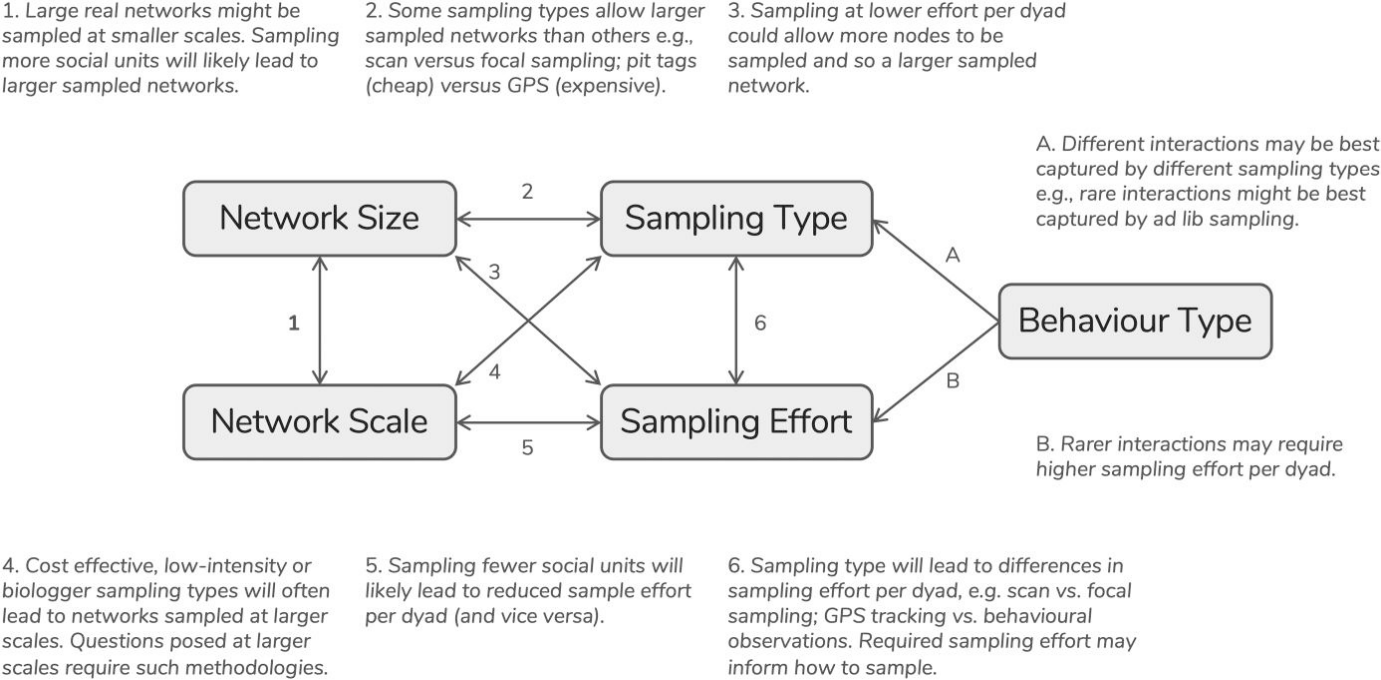
Links between the 5 key challenges faced when comparing social networks. This figure is not meant to be exhaustive, but rather to highlight some of the many ways in which the challenges we outline can interact.

While we present these challenges independently here for clarity, it is key to note that these challenges are closely linked (Fig. 2). For example, the size of the *realised network* may influence the scale at which sampling is undertaken, which can then in turn influence the size of the *observed network*.

### 1. Differences in behavior type

The first factor determining the comparability of networks is the type of behavior used to construct them. For most comparative analyses, networks compared should be constructed on behaviors with similar biological functions, therefore, reflecting similar *social preferences* or representing *realised networks* with similar outcomes (Box 2). While it might seem obvious that an affiliation network based on huddling should not be directly compared to an agonism network based on physical aggression, determining which behaviors *can* be compared often requires more nuanced considerations. For example, various behaviors have been used to quantify affiliative social relationships in different studies and species, ranging from direct interactions such as grooming and allopreening, to spatial associations and co-membership of a group (Smith-Aguilar et al. 2018; Webber and Vander Wal 2019). Whether these behaviors can be considered to represent the same biological function is contingent on the research question and the biology of the study species to be compared (Carter et al. 2015; Farine and Whitehead 2015). In addition, the same behavior can serve different functions in different systems and might reflect different information depending on the context. For instance, pairs of animals sitting within a 5 m range of one another could be indicative of a close association in wild populations, whereas that same distance might not carry the same information in a captive population where individuals have less space over which to spread.

Box 2: Latent layers and multilayers in social network analysis There are some superficial similarities between our *latent layers framework* and the concept of multilayer networks (Kivelä et al. 2014; Finn et al. 2019). In fact, it would be possible to represent the *observed*, *realised*, and *social preference networks* as layers of a multiplex network. But given that the latent layers represent abstractions of the same social structure, analysing them as a multiplex network is unlikely to be helpful. However, the latent layers framework could equally apply to multiplex networks. For example, in a case where a researcher was studying multiple social behavior types together (eg, grooming, nuzzling and greeting interactions), then each layer of our framework could be multiplex rather than single layer networks, with an observed multiplex network and a realized multiplex network. In this case it is interesting to consider whether the *social preference network* is best described as multiplex (representing different preference networks for different types of social interactions) or as a single layer (the same preferences combining with constraints in different ways to generate the realized network).

Moreover, behaviors will differ in how strongly individuals are *constrained* in realizing their *social preferences*. For example, certain behaviors like grooming or biting are often difficult to direct at more than one partner at a time. In contrast, individuals can sit near or vocalize to multiple partners at once, so that these types of behaviors are likely to be less constrained by limitations related to social preferences. Similarly, environmental constraints, such as spatial barriers, are more likely to impact behaviors that involve physical contact than behaviors that do not. Different behaviors are also influenced by *sampling biases* to different extents, so that an *observed network* of one behavior might better represent the *realised network* than another behavior. Rare or less visible behaviors tend to be more heavily affected by *sampling biases* (Bateson and Martin 2021). Moreover, the degree of *sampling bias* can vary for the same behaviors depending on the system or context. For example, recording aggression in a terrestrial species may be easier than in an aquatic one. Consequently, a smaller proportion of interactions may be observed for the aquatic species compared to the terrestrial one.

### Considerations for comparing networks of different behavior types

Any comparative study whose question depends on comparing “like to like” will need to carefully evaluate whether the networks are constructed based on behaviors with comparable biological functions and facing similar *sampling biases* and *constraints* in their given context (or whether differences can be accounted for in the analyses). This is important because behaviors should either reflect similar *social preferences* or represent a *realised network* with similar outcomes. Determining which behavior types can be reliably compared is a critical first step in comparative social network analysis, which requires thoughtful consideration tailored to the specific research question and informed by knowledge of the species’ biology under study.

In our fish and bird example, we have data on multiple social behaviors for each species: cooperative predator mobbing and swimming in parallel in the fish, cooperative nest-building and preening in the birds. Of these, we choose to use predator mobbing in fish and nest-building in birds, as they are functionally comparable, both reflecting cooperative social interactions that likely involve partner investment decisions.

#### 2. Differences in sampling effort

Sampling effort can significantly impact how reliably an *observed network* represents the *realised network*, with greater effort improving reliability (Croft et al. 2008; Whitehead 2008; Farine and Strandburg-Peshkin 2015; Shizuka and McDonald 2015; Franks et al. 2021). Accounting for uncertainty in estimated network metrics is important for any social network analysis but becomes especially crucial when comparing networks constructed with varying sampling efforts. These networks inherently differ in how reliably the observed strength of dyadic connections, ie edge weights, represent the underlying actual edge weights. Failing to account for this uncertainty might lead to wrong conclusions. For instance, in a network constructed based on just one hour of observation, a dyad may appear to spend most (or none) of their time together. Yet, this estimate could be a highly uncertain representation of this dyad's connection in the *realised network*. Extending to 100 hours of sampling effort may provide a more accurate estimate of the dyad's edge weight in the *realised network* and would lower the level of uncertainty around that estimate. Comparing a low observation effort network to a high observation effort network might falsely suggest that individuals in the low observation network spend more (or less) time together compared to those in the high observation network, but in reality, the difference is due to sampling effort rather than actual differences in behavior. One effective solution to address the challenge of variable sampling effort when comparing networks is to use models that estimate the *realised network* based on the *observed network* as a latent structure, with an explicit degree of uncertainty (Box 1).

#### Considerations for comparing networks of different sampling effort

When comparing networks, researchers should account for differences in sampling effort, which can strongly impact how well an *observed network* represents the *realised network*. An effective solution to do so is to consider the *realised network* as a latent structure, which is estimated with a degree of uncertainty, determined by the observation effort. Recent frameworks provide tools to estimate the *realised network* from an *observed network*, while explicitly estimating uncertainty in the estimated latent network based on sampling effort (Box 1).

In our fish and bird example, the networks have been sampled in different ways. For the fish, 2 datasets are available: one based on high-intensity sampling over a single month and another based on lower-intensity sampling conducted over several months. For the birds, data were collected over several months. Because our research question focuses on more stable social relationships rather than short-term interactions—and to ensure better comparability with the bird network—we decide to use the longer-term, lower-intensity fish dataset. The fish network includes many more individuals than the bird network, so even though the study duration and rate of interaction in our behaviors of interest is similar across both species, the per-individual sampling effort is comparatively lower in the fish. To account for this difference, we use BISoN (Hart et al. 2023), which allows us to quantify uncertainty in estimated relationships based on variation in social effort and to carry this (difference in) uncertainty through into downstream inference.

#### 3. Differences in sampling type

Many challenges are associated with comparing networks constructed using different sampling methodologies (Albery et al. 2024; Mielke et al. 2025). The 2 we focus on here are differences in sampling protocol and differences in how data are summarized into edge weights.

Different sampling protocols can impact how an *observed network* relates to the *realised network*. Some of the most commonly used sampling protocols include focal continuous sampling (recording social interactions and/or associations, referred to collectively as “interactions' from here on, that include a given individual for a set amount of time), group scan sampling (recording the social interactions of each individual in a group instantaneously at regular time intervals) and gambit of the group sampling (recording group compositions during repeated surveys, where individuals observed in the same group are taken to be associating with each other; Altmann 1974; Whitehead 2008). More recently, technologies such as camera traps, biologgers, or drones have been used to observe and record animal behavior remotely (Webber and Vander Wal 2019; Smith and Pinter-Wollman 2021). Each of these sampling protocols have inherent biases in the interactions that are recorded (Altmann 1974; Mielke et al. 2025). For instance, focal continuous sampling provides rich, detailed data on the focal individual and its social partners for a given time but overlooks social interactions between all other individuals during that time. Similarly, sampling using biologgers is often limited to a subset of individuals in the group because of their high cost and deployment schedules (eg, not all animals can have a biologger deployed at the same date). Sampling the whole group at regular time intervals (using scan sampling or camera traps), on the other hand, can capture the overall occurrence of interactions across group members to a greater extent but misses interactions occurring between sampling intervals.

A second challenge is that networks are built based on different measures to quantify the strength of dyadic connections (edge weights), which are not always directly comparable and can even sometimes represent different aspects of the *social preference network*. Social interactions are typically collected by recording a count of the number of interactions observed or the duration of each interaction observed (Martin and Bateson, Altmann 1974). Which of these methods are used is determined, in part, by the selected sampling protocol, although certain protocols allow for the collection of multiple types of data. Edge weights may then be represented as (1) the *rate* of social interactions per unit of time (a count of the number of social interactions recorded divided by observation time), (2) the *proportion* of time 2 individuals spend engaged in a social interaction (the total duration of social interactions recorded divided by observation time), or (3) the *probability* of a social interaction occurring between 2 individuals within a specific time frame (a count of the number of samples during which an interaction is recorded divided by the total number of samples). Probabilities and proportions are both unitless measurements bound between zero and one. Rates, on the other hand, are the expected number of events per unit time (eg associations per hour), with a lower bound of zero and no upper bound. Because the probability of being in a particular state at a point in time is equal to the proportion of time spent in that state, a unit increase in a probability is equivalent to a unit increase in a proportion, allowing direct comparisons between probabilities and proportions, all else being equal. However, comparing probabilities or proportions to rates is not as simple, as there is no natural way to interpret these 2 distributions of data on the same scale. This also relates to a more conceptual point: how often individuals interact with a given partner (rates), and how much of their social time they spend on a given partner (proportions/probabilities) are meaningful aspects of the *social preference network*, which do not necessarily carry the same information (Dunbar 1976). For instance, animals might interact frequently for brief amounts of time with certain types of partners and interact less often but for long amounts of time with others.

Selecting an appropriate interaction index can help mitigate issues related to sampling type to a certain extent. Various indices have been devised to address different *sampling biases*, making networks built from different sampling protocols more comparable (Franks et al. 2010). Moreover, some network metrics, such as unweighted network metrics, or metrics that are expressed relative to the mean edge weight of the group, are less sensitive to differences in sampling type. In addition, using a standardization such as Z-scoring makes edge weights (or derived measures of network structure) interpretable in terms of standard deviations, thereby making rates and proportions/probabilities more comparable. However, these approaches do not account for the different *sampling biases* inherent to these distinct sampling processes, which may introduce disparities between an *observed network* and the *realised network*. Nor do they account for how different measures of edge weights might capture different aspects of the *social preference network*.

Alternatively, mixture models can be used to identify similar interaction levels in the *observed network*, creating categories of individuals that share strong, intermediate or weak social relationships (ie estimating the social preference network; Weiss et al. 2019; Ellis et al. 2021). These categories are robust to variation in sampling type and can therefore readily be compared. A final solution is to integrate the sampling process into analytical models that estimate the *realised network* based on an *observed network* (Box 1). Bayesian models have been developed to reconstruct latent networks by explicitly incorporating assumptions about how the sampling process impacts the relationship between an *observed network* and the *realised* or even the *social preference network* (Young et al. 2020).

#### Considerations for comparing networks of different sampling type

When comparing networks derived from various sampling methods, 2 main factors need to be considered: (1) biases in the recorded social interactions due to differing sampling protocols, and (2) differences in how edge weights are measured. Although employing suitable indices and Z-scoring edge weights can alleviate some of these concerns, these approaches do not account for the disparities between an *observed network* and the *realised* or *social preference network* that differences in sampling can generate (Fig. 1). Recent methodological advancements, including mixture models and models that estimate the *realised network* as a latent structure while accounting for the sampling process offer promising avenues to navigate these challenges effectively (Box 1).

In our fish and bird example, the behaviors we chose to build the networks on—predator mobbing in fish and nest-building in birds—were collected using different methods. Predator mobbing was recorded using group scan sampling, yielding proportions of scans in which individuals co-occurred during mobbing events, while nest-building was recorded through focal continuous sampling, providing durations of each nest-building event involving the focal subject. Despite these methodological differences, we prioritized the functional comparability of behaviors when selecting them for our comparative study and proceeded with our analysis accounting for differences in sampling type as best we can. To assess the number of social relationships formed by each individual, differences in sampling type are not very problematic, as we are primarily interested in whether an individual did or did not interact with each potential partner. However, differences in sampling type can have a stronger influence on the estimated relationship strength with each partner. To address this, we use mixture models to identify distinct clusters of relationship strength in the observed data—a method that is more robust to variation in sampling type.

#### 4. Differences in network size

Network size, or the number of nodes in a network, varies substantially in animal societies ranging from only a few individuals to large assemblages of several hundred individuals (Webber and Vander Wal 2019). This variation is shaped in part by differences in social organization, dispersal patterns, ranging behavior, and/or territoriality (Kappeler 2019). In addition, *observed networks* can include different subsets of the underlying *realised network*, driven by researchers’ choices regarding who is observed (for instance focusing only on adults or on habituated individuals; Richardson and Cords 2025). Observed network size therefore depends on the features of the social system being investigated (which determine *realised network* size) as well as the decisions made by researchers when designing their study (which determine what subset of the *realised network* is observed). Here we consider what differences in the realised network sizes mean for the comparability of network structures. We explore the effects of sampling different subsets of *realised networks* in the next section (*Differences in network scale*).

**Fig. 3.**
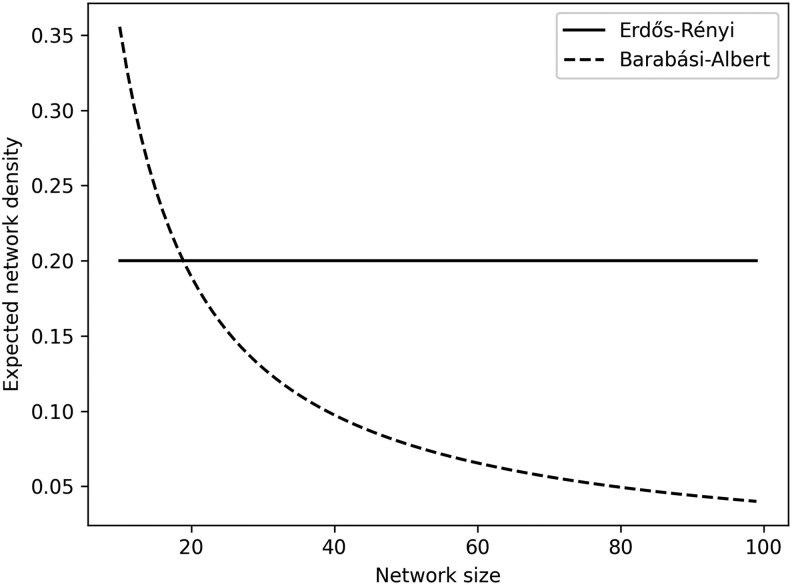
Measures of network structure can be influenced by network size in different ways, depending on the underlying processes that generate the network. In this example we compare how unweighted network density is influenced by network size for networks generated from 2 different processes. If individuals choose their interaction partners at random (‘random attachment”, captured by the Erdős-Rényi model, Erdős and Rényi 1959) with a fixed probability (eg each pair of individuals has a 20% chance of interacting) then the network density stays the same regardless of the size of the network. In contrast, if individuals preferentially interact with their most popular groupmates and each individual contributes with a fixed number of edges (‘preferential attachment”, captured by the Barabási-Albert model, Barabási and Albert 1999), then the density of the network declines exponentially with its size.

Comparing networks of different sizes is a complex challenge because network size can greatly influence other aspects of network structure in non-trivial ways. The effect of network size on social structure depends on the nature of the underlying process generating the network (Box 1, Boccaletti et al. 2006; Hobson, Silk et al. 2021) and is often not the same for different measures of network structure (Fig. 3; Anderson et al. 1999; Naug 2009). Therefore, controlling for network size (eg dividing network metrics by network size, or adding network size as a control predictor in analyses) does not always fully account for size effects, and may even introduce biases (Deffner et al. 2022).

**Fig. 4.**
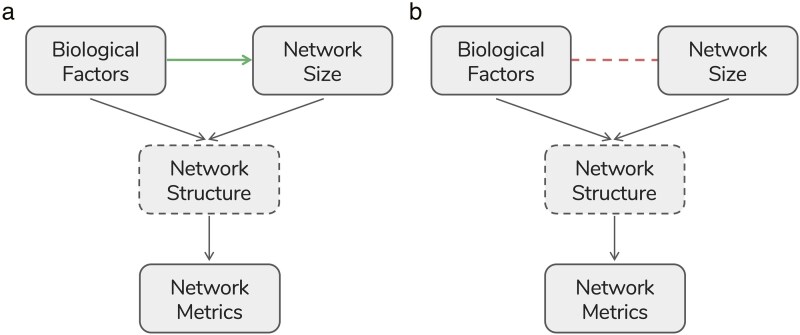
Potential causal mechanisms linking network size to structure. Note that “network” here refers to the realized network. The solid arrows represent causal relationships and the dashed lines indicate a potential non-causal association. The dashed boxes indicate that the structure of the realized network is a latent, unobservable variable, which can be quantified, but not entirely captured by a network metric, ie a measure of network structure. In a), biological factors affect network structure partially via network size (ie there is a causal relationship between biological factors and network size, illustrated with the green arrow), and controlling for network size would mask some of the influence of biological factors on network size. In b), biological factors do not directly drive network size, but through exogenous variables, biological factors and network size may be correlated (ie there is a potential non-causal association between biological factors and network size, illustrated with the red dashed line). In this case, unless network size can be effectively controlled for, the overall impact of biological processes on network structure cannot be estimated, as network size is a possible confound. In both cases, potential confounds act on the network structure itself, not on the network metric. As long as the chosen network metric is an accurate quantification of network structure, potential challenges do not lie with the metric, but with underlying causal assumptions.

The first question to consider when comparing networks of different sizes is *whether it is appropriate* to control for network size. Biological factors may influence key aspects of network structure through their effects on network size (at least in part; Shizuka and McDonald 2015; Kawam et al. 2025). Network size can therefore be considered an important feature of social structure itself. For example, if an individual's risk of being infected by a pathogen depends on its number of social partners, then being in a larger group can be part of the cause of a higher exposure risk. In these cases, conditioning network comparisons on network size would mask effects of biological importance (Fig. 4a). However, for other research questions, it may be necessary to condition on network size to make meaningful biological comparisons. For example, to test the hypothesis that forest-living species (typically living in smaller groups) have denser social networks than those in open habitats (typically living in larger groups), conditioning on network size is necessary to demonstrate that habitat influences network structure (density) in a manner that is not solely driven by the relationship between habitat and network size (Fig. 4b).

The next question to consider is *how to control* for differences in network size: even when controlling for network size is appropriate for a specific question, doing so in a way that correctly removes size effects can be difficult. The relationship between size and network structure depends on the process that generates the network, and these processes are often unknown (Box 1, Brask et al. 2023). For instance, unweighted network density (the ratio of actual to potential connections in a network) is differently impacted by network size, depending on the process that generates the network (Fig. 3). If individuals choose their interaction partners at random with a fixed probability, then network density stays the same regardless of the size of the network. In contrast, if individuals preferentially interact with their most popular groupmates and each individual forms a fixed number of relationships, then the density of the network declines exponentially with its size. Similarly, the strength of social relationships can depend on network size in different ways. If individuals get more partners when the network is larger but are restricted in the amount of time they have available to socialize, relationship strength will decrease with network size. In contrast, relationship strength can be independent of network size if individuals keep a constant number of partners regardless of network size, or if they get more partners and also increase the amount of time they spend socializing (so that they can spend the same amount of time with each of their partners even when their number of partners increases). Each of these scenarios require different approaches to correctly remove the effect of network size (Hobson, Silk et al. 2021). That is, if the networks to be compared have emerged from different generative processes, then correctly controlling for size may involve a different control procedure for each network.

Properly accounting for network size in comparative network analysis is often difficult, if not impossible, unless valid assumptions are established about the underlying processes that generate the network (Hobson, Silk et al. 2021). Understanding *generative processes* in animal social networks is an area that still needs substantial methodological progress (Box 1, Brask et al. 2023), but one where the *latent layers framework* may be particularly helpful. By explicitly considering the *social preferences* driving a given network, we can better understand the expected relationship between network size and relationship strength within the system. This in turn, can inform the design of simulations to predict how network structure varies with changes in size and guide decisions on whether and how to account for network size in subsequent analyses.

#### Considerations for comparing networks of different size

When comparing networks, differences in network size are almost inevitable. Whether and how to account for differences in network size is a long-standing challenge in network science, and solutions are often context-dependent (Croft et al. 2008; Whitehead 2008). When network size is central to how network structure relates to biological variables of interest, conditioning on network size could mask important effects. When biological factors do not directly influence network size, or when biological effects that go through network size are not of interest, conditioning on network size is warranted. To condition properly requires an understanding of the *generative processes* (ie the *social preferences* and *constraints*) underlying the networks, to understand the relationship between network size and measures of network structure (Box 1). As this information is often lacking, we suggest restricting comparisons to networks of similar size, or where it is reasonable to assume similar underlying *generative processes* and therefore similar relationships between network size and other components of network structure.

In our fish and bird example, the fish network includes considerably more individuals than the bird network—a difference that reflects real biological variation in group size. One consequence of this is that each fish has been observed less than each bird, something we accounted for by incorporating observation effort into our analyses (see *Considerations for comparing networks of different sampling effort*). Whether to account for group size beyond this methodological difference is a complex issue, as our central research question—whether individuals tend to invest in many relationships or a few strong ones—is intrinsically linked to network size. We therefore choose to run our models both with and without controlling for network size: once to understand how much variation in social structure remains after accounting for network size, and once to capture the full extent of variation in social structure, including that which may reflect meaningful biological differences driven by differences in network size.

#### 5. Differences in network scale

**Fig. 5.**
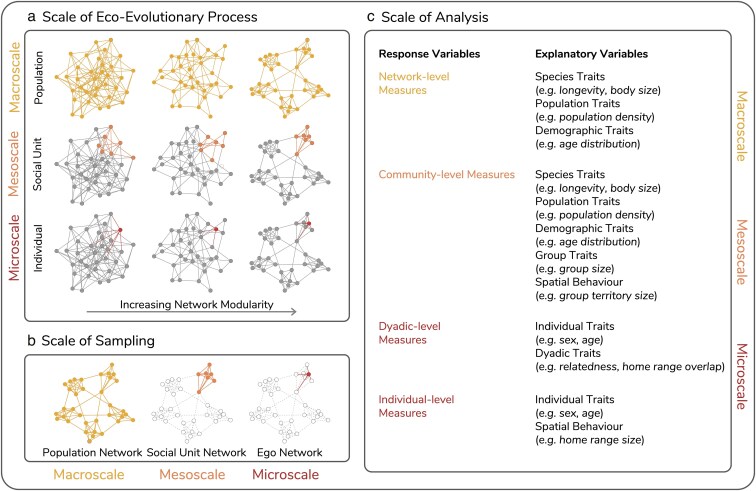
An illustration of the challenges of scale in social network analyses. a) Social network analyses address questions related to various ecological and evolutionary processes for which different scales of network structure are important. For example, processes like disease transmission typically operate at the population level, while patterns of cooperation or competition often emerge within smaller social units, such as groups or communities. Networks can vary considerably between species across these scales. For instance, in species that cluster into groups, network modularity (illustrated), is higher at the population level (macroscale) than the social unit-level (mesoscale). Nodes and edges in color represent the eco-evolutionary process of interest at different scales. b) Research teams make decisions about the scale at which to sample networks based on biological properties of their study system, their research question and time and budget constraints. Nodes and edges in color represent the parts of the entire networks that were sampled at different scales. c) Research teams make decisions about the scale of analysis, focusing on measures that may capture the structure of their sampled network as a whole down to measurements that quantify the network position of single individuals. Any mismatches in the scales at which the eco-evolutionary processes of interest operate, and the scales at which networks are sampled and analysed can generate challenges for subsequent comparative analyses.

Networks can be studied at different social and spatial scales, depending on the ecological or evolutionary process of interest and limitations in data collection. For instance, some studies may focus on a single group, while others examine the entire population; similarly, some studies include all individuals, whereas others sample specific subsets of individuals based on traits such as sex or age (Richardson and Cords 2025). As a result, *observed networks* can represent samples of the *realised network* at different scales, which can introduce biases in comparative analyses if they are not conducted with appropriate caution (Fig. 5).

The extent to which comparing across network scales poses a challenge depends on the scale of analysis required to answer the question of interest (Fig. 5). Measures of network structure that are being compared can vary from measures of the connectedness of individuals (microscale), to measures of the structure of the whole network (macroscale; Hobson et al. 2019). When comparing networks, it is important to think carefully about the scale of interest for the research question, and whether that scale is the same or different for the study systems to be compared (Hobson et al. 2019). Just as for behaviors (see the previous section *Differences in behaviour types*), the same scale does not necessarily represent the same biological phenomenon in different systems. Comparing networks at different scales might also reveal differences that would not appear at one scale alone (McDonald and Shizuka 2013). For instance, individuals of 2 different species might form the same number of relationships on average, but the overall network density might be very different if individuals from one species live in small groups and those from the other species live in large groups, or if one species was sampled at the group-level and the other species at the population-level, or if one study focused solely on adult females and the other on adult males. Moreover, properties at one scale can influence properties at a different scale and vise versa (Cantor et al. 2021).

Network metrics at the level of the social unit or population (meso- and macroscale, respectively) are likely to be particularly susceptible to differences in the scale of sampling between datasets (Ogino et al. 2023). For example, comparing network density (a macroscale measure) from a single social group with that of an entire population can be misleading because the scale of observation affects network density. Individuals within a single group are typically more strongly connected to each other, leading to higher density, while a population-level network often includes multiple loosely connected groups, resulting in lower overall density. In contrast, microscale (individual-level) metrics of networks are often less affected by scale and will be more reflective of values in the *realised networks* (including non-sampled individuals). However, this is not necessarily true, and depends on the *generative processes* underlying the network structure and the specific choice of individual-level metric. For example, in species where individuals interact with others outside their group, an individual will have much higher betweenness centrality when a population- rather than group-level network is considered.

In some cases, it may be possible to compare networks sampled at different scales by sub-sampling from the network sampled at a larger scale, but this process is untested and fraught with complex decisions on how best to subsample (eg, see the previous section *Differences in network size across networks*). Another potential solution is to estimate the *realised network* as a latent structure from an *observed network* by using imputation to “fill in” missing parts of networks sampled at a smaller scale (Box 1, Young et al. 2020). Doing so requires information about the *generative processes* underlying the network, which currently are not well developed (Box 1). For example, in many studies focused on within-group networks, interactions with members of other groups may not be recorded or may occur so infrequently that they remain unobserved. This means that we know little about the *social preferences* and *constraints* generating networks beyond the scale of the group. In these cases, it would be impossible to reliably infer the *realised network* beyond the scale of the original study.

#### Considerations for comparing networks of different scale

Networks can be sampled across various scales, and observed networks representing different scales usually cannot directly be compared. One solution is to use methodological advancements that can impute missing data to reconstruct the *realised network* at a larger scale. However, reliable imputation requires an understanding of the *generative processes* underlying the networks, including the processes that drive interactions beyond the scale that was sampled (Box 1). As this understanding is most often lacking, we suggest that comparisons should be restricted to cases where it is reasonable to assume networks have been sampled at a similar scale.

In our fish and bird example, the networks differ in the scale at which they are sampled. The fish network represents the entire local population, while for the birds, networks were originally built for each individual nest of cooperative breeders. However, because we have data from most nests in the population, we choose to combine these to construct a single network representing the entire breeding population, to allow for more direct comparability with the fish network.

## Discussion

Comparative social network analysis offers huge potential to answer fundamental questions in ecology and evolution, but this approach comes with a set of major challenges that are yet to be fully resolved (Albery et al. 2024). In this paper, we present the *latent layers framework* that explains how observed animal social networks are related to the latent social structures and processes of interest to researchers. We then outline 5 key challenges in comparative analyses of social networks. Using the *latent layers framework* as a base, we consider how these challenges can lead to erroneous conclusions, and we discuss the current state of solutions to mitigate these challenges. By doing so, we have aimed to offer guidance on factors to consider before embarking on comparative social network analyses and to inspire further developments of methodological tools that enable these types of analysis to be conducted robustly and to their full potential.

In addition to giving potential solutions for each specific challenge in the sections above, our summary of our overall current guidance for comparative network analysis is to

- Identify the latent network layer to which your research question applies (Fig. 1)—are you interested in the *realised network*, or the *social preference network*? This will shape your analytical decisions going forward.
- Be mindful and clear about the limitations of any approach used. Different networks may have been affected by different inherent *constraints* and *sampling biases*, and this can influence observed differences between them.
- Consider whether *constraints* and *sampling biases* should be treated as noise, signal or part of the causal pathway for your research question. For example, if the aim is to understand individual strategies given ecological or social limitations, *constraints* should be explicitly modeled as part of the *generative process*. Similarly, differences in network size can generate *sampling biases*, in which case they should be accounted for, or be a major driver of the difference of interest, in which case accounting for size would mask relevant differences.
- Consider how differences between networks to be compared can be addressed analytically. Bayesian methodological developments offer promising solutions, by estimating the *realised*, or even the *social preference network*, while explicitly accounting for key differences in compared networks (Box 1). Maintain uncertainty in the estimates when moving between network layers and propagate this uncertainty into subsequent comparisons.
- Consider the *generative processes* that link the latent networks to the observed network for different sampling methodologies and/or datasets (Box 1). Understanding these processes can help identify how to account for methodological differences to make different networks comparable or can help (careful) imputation of missing information.

The *latent layers framework* highlights future theoretical and methodological work that will be central to facilitating analyses that make inferences about latent network layers (both for comparative network analyses and for network analysis more generally). Moving between network layers necessitates detailed knowledge of the key processes that influence the emergent structure of animal social networks (Box 1). While such knowledge is available for some particularly well-studied species, a combination of theoretical modeling and empirical analyses will often be necessary to identify patterns that can be used to pinpoint the generative processes underlying animal social networks. New statistical tools will then be required to efficiently estimate the latent networks, as well as to tailor existing observation models (eg from capture-recapture models) to social network contexts (Silk and Gimenez 2023). These steps forward will benefit greatly from interdisciplinary collaborations between behavioral ecologists, statisticians, and network scientists (Brask et al. 2021).

Through decades of research on animal social behavior, a substantial and growing body of social network data has been collected across a wide range of animal species, capturing rich variation in social structure within and between populations. Combined with advances in analytical methods and a shift toward more collaborative research practices, the field is now well positioned to move beyond single-species studies and begin addressing broader, comparative questions about the drivers of variation in social systems across the animal kingdom. With further development, comparative social network analysis could become an accessible, reliable, and powerful approach to answer long-standing questions in ecology and evolution. We hope this paper motivates researchers to adopt comparative approaches to social structure and equips them with the insights needed to meet the challenges ahead. Good luck!

## Glossary

**Social preference network**: A network that represents individuals’ preferences for how frequently they would like to interact/associate and with whom.

**Realized social network**: A network representing the full pattern of social interactions or associations among individuals.

**Observed social network**: A network that researchers typically work with, built from the interactions or associations that were observed and recorded.

**Sampling biases**: Systematic differences in how well certain individuals or interactions/associations are observed and recorded. Sampling biases can arise from factors such as uneven observation effort across individuals, and more observations in better visible or accessible areas, or only during certain times of the day or year.

**Constraints**: Factors that prevent individuals from realizing their social preferences. Constraints can arise from several factors, including individual limitations like energetic constraints or trade-offs in time and resource allocation, incompatible preferences between potential partners, or social factors (such as dominance hierarchies or kinship structures) and environmental barriers (such as spatial distance or rivers) that restrict access to certain partners.

**Behavior type**: The specific social behavior used to construct a network.

**Sampling effort**: The intensity or duration of data collection per individual or group.

**Sampling type**: The data collection approach (eg, focal sampling, group scans, biologgers) used to record social interactions.

**Network size**: The total number of individuals in a network.

**Network scale**: The social or spatial level at which a network is sampled and/or analysed (eg at the level of the whole population or of the social group).

## Data Availability

This paper does not contain any data.
